# Targeted *in vitro *and *in vivo *gene transfer into T Lymphocytes: potential of direct inhibition of allo-immune activation

**DOI:** 10.1186/1471-2172-7-26

**Published:** 2006-11-10

**Authors:** Ashwani K Khanna, Mandeep R Mehra

**Affiliations:** 1 Department of Medicine, Division of Cardiology, University of Maryland, Baltimore, MD-21201 USA

## Abstract

**Background:**

Successful inhibition of alloimmune activation in organ transplantation remains one of the key events in achieving a long-term graft survival. Since T lymphocytes are largely responsible for alloimmune activation, targeted gene transfer of gene of cyclin kinase inhibitor p21 into T cells might inhibit their aberrant proliferation. A number of strategies using either adenoviral or lentiviral vectors linked to mono or bispecific antibodies directed against T cell surface markers/cytokines did not yield the desired results. Therefore, this study was designed to test if a CD3promoter-p21 chimeric construct would *in vitro *and *in vivo *transfer p21 gene to T lymphocytes and result in inhibition of proliferation. CD3 promoter-p21 chimeric constructs were prepared with p21 in the sense and antisense orientation. For *in vitro *studies EL4-IL-2 thyoma cells were used and for *in vivo *studies CD3p21 sense and antisense plasmid DNA was injected intramuscularly in mice. Lymphocyte proliferation was quantified by ^3^H-thymidine uptake assay; IL-2 mRNA expression was studied by RT-PCR and using Real Time PCR assay, we monitored the CD3, p21, TNF-α and IFN-γ mRNA expression.

**Results:**

Transfection of CD3p21 sense and antisense in mouse thyoma cell line (EL4-IL-2) resulted in modulation of mitogen-induced proliferation. The intramuscular injection of CD3p21 sense and antisense plasmid DNA into mice also modulated lymphocyte proliferation and mRNA expression of pro-inflammatory cytokines.

**Conclusion:**

These results demonstrate a novel strategy of *in vitro *and *in vivo *transfer of p21 gene to T cells using CD3-promoter to achieve targeted inhibition of lymphocyte proliferation and immune activation.

## Background

Aberrant T lymphocyte proliferation is a key mediator of alloimmune activation in organ transplantation. Therefore, T cells are the key targets for direct transfer of genes that could inhibit their proliferation and alloimmune activation. A number of strategies using either adenoviral or lentiviral vectors linked to mono or bispecific antibodies directed against T cell surface markers/cytokines did not yield the desired results [1-4]. The efficacy of a CD3 promoter-p21 chimeric construct to transfer p21 gene to T lymphocytes was tested. Cyclin kinase inhibitor p21 is a potent inhibitor of lymphocyte proliferation and inflammation [5,6]. Inflammation is realized as one of the key mediators of a number of diseases associated with aberrant cellular proliferation including alloimmune activation and organ transplant rejection. [7-12]. Transfer of genes into T cells remains a critical step to achieve successful therapeutic strategies for such diseases. It is also clear that resting T cells, which make up most of the circulating T-cell pool *in vivo*, cannot be specifically and efficiently transfected due to the presence of other immune cells in the same milieu. The direct adenoviral and lentiviral vectors mediated gene transfer to T cells was not successful and the modification and coupling of these vectors with antibodies to CD3 and other T cell surface receptors permitted some limited success [1-4,13-16]. Interestingly, expression of cyclins and cyclin-dependent kinases and pro-inflammatory cytokines is increased during T lymphocyte proliferation [5]. Therefore, an effective control of T cell proliferation by regulating the expression of cyclins would potentially inhibit alloimmune activation and inflammation. Cyclin kinase inhibitor p21 (here on after only p21) is one of the most potent cyclin kinase inhibitor and therefore has potential to control the expression of cyclins and T cell activation. The inhibition of cyclin dependent kinases and cyclins by p21 will prevent excessive proliferation and prevent alloimmune activation by the direct inhibition of T lymphocyte proliferation.

At the present time one of the most effective means to inhibit T lymphocyte activation and alloimmune activation is with the immunosuppressive agents; cyclosporine (CsA), tacrolimus (TAC) and sirolimus (SRL). However, the long-term usage of these drugs leads to number of side effects including nephrotoxicity, malignancy and viral infections [17-19]. We have demonstrated that the T cell inhibitory effects of CsA, TAC and SRL are in part mediated by the induction of p21 [20-22]. Our studies have also demonstrated *in vitro *and *in vivo *over-expression of p21 in lymphocytes results in decreased response to mitogenic stimuli and greater sensitive to the inhibitory effects of CsA [23]. We have also shown that the over expression of p21 in the recipients of rat heart transplant recipients resulted in increased graft survival via the inhibition of mRNA expression of pro-inflammatory cytokines in allografts and lymphocytes [5]. Most significantly, we recently demonstrated that the recombinant p21 protein localizes into the nucleus, interacts with transcription factors and inhibits lymphocyte proliferation and markers of inflammation [24].

Therefore, the present study was designed to explore *in vitro *and *in vivo *gene transfer of p21 targeted to T cells using chimeric CD3 promoter-p21 constructs. For *in vitro *studies, mouse thyoma cell line EL4, which constitutively express T cell growth factor IL-2 were used. The *in vivo *effects were evaluated by intramuscularly injecting CD3-p21 sense and antisense plasmid DNA in mice. The results demonstrate the feasibility p21 gene transfer targeted to T cells with the CD3-p21 chimeric constructs with its biological consequences that is modulation of lymphocyte proliferation and alloimmune activation.

## Results

### CD3Sensep21 and CD3AntiSensep21 constructs

**Figure 1:**
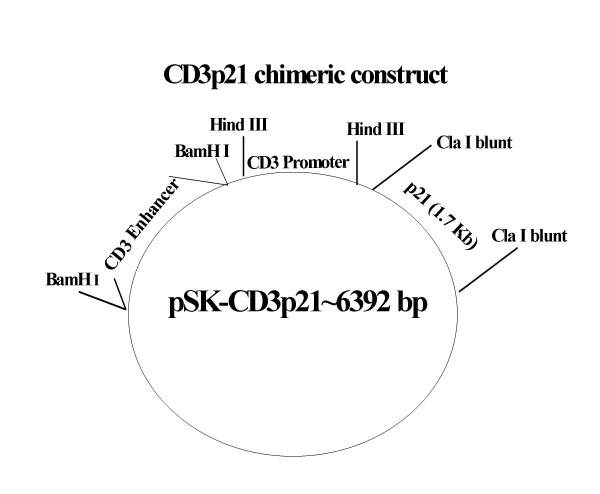
**Construction of CD3-p21 chimeric plasmid conctruct**. A complete vector structure of pSK-CD3-p21 chimeric plasmid is shown. The restriction enzyme sites for CD3 promoter, enhancer and p21 gene are also shown. Plasmid TGE-CD3 contains the murine T cell-specific delta enhancer and promoter. CD3 promoter was cut with Pst I and blunted with T4 DNA polymerase. HindIII linkers were ligated to the fragment and, following HindIII digestion, cloned into the HindIII site downstream of the enhancer region. The p21 gene was excised with restriction enzymes KpnI and XbaII, blunted with DNA polymerase I, large (Klenow) fragment, and cloned into the blunted Cla I site just downstream of the CD3 promoter in the enhancer/promoter SK construct.

A diagrammatic presentation of CD3p21 chimeric construct with sequences of CD3 promoter, enhancer and p21 genes in pSK plasmid is shown in Figure 1. Chimeric constructs with p21 in the sense and anti-sense orientation are designated as CD3sense p21 and CD3antisensep21, respectively.

### Efficiency of Transfection

#### a) In vitro

Transfection efficiency was expressed as the percentage of the total cells in the gated region that displayed green fluorescence, i.e. expressed in the pEGFP-p21 plasmid. A mean of 76% ± 6 EL4-IL-2 cells were found to be positive from three consecutive transfection experiments, compared to completely negative for green fluorescence of untransfected cells.

#### b) In vivo

**Figure 2:**
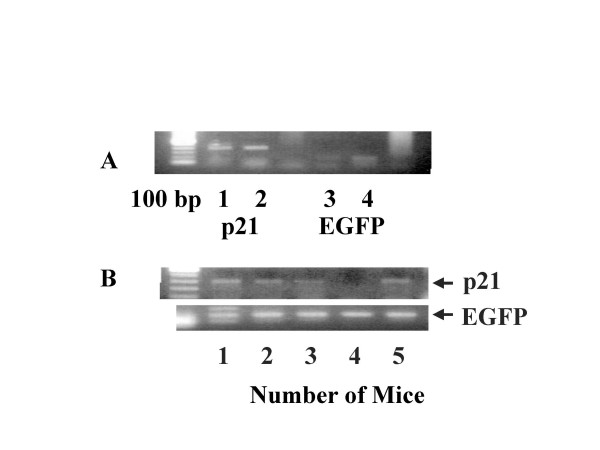
**Construction and efficacy of p21-EGFP construct**. p21-EGFP constructs were prepared by ligating p21 gene construct in Multiple Cloning Site of EGFP vector. Colonies were picked and amplified for p21 and GFP gene expression. **A**. The results of PCR amplification of two representative colonies are shown. DNA from colony #2 (**lanes 2 and 4**) exhibits stronger gene expression for both p21 and GFP compared to colony #1 (**lanes 1 and 3**). More DNA was prepared from colony #2 and used to assess *in vivo *efficiency of transfection using intramuscular injection. **B**: Efficacy of intramuscular injection of p21-GFP plasmid DNA is shown as p21 and GFP mRNA expression in splenocytes individually from 5 mice.

Intramuscular injection of p21-EGFP plasmid DNA resulted in increased expression of p21 and GFP mRNA in splenocytes (only tissue studied). The expression of p21 mRNA was seen in 4/5 mice and GFP mRNA in 5/5 mice (Figure 2B).

### Targeted p21 overexpression inhibits IL-2 expression by EL4 thyoma cell lines

**Figure 3:**
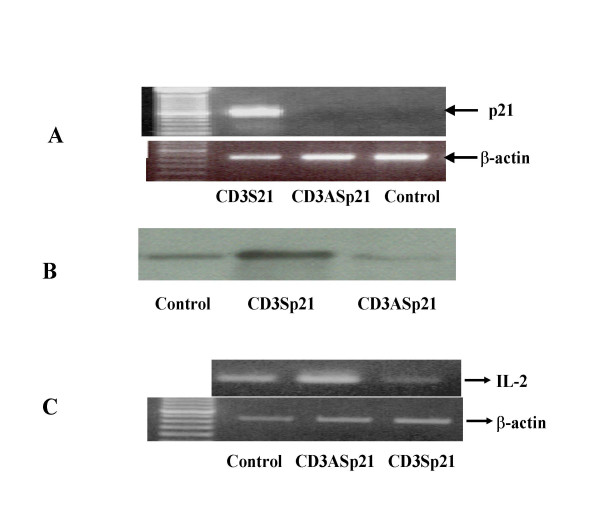
**Efficacy of CD3-p21 chimeric plasmid DNA**. The efficacy of CD3-p21 plasmid DNA in mouse Thyoma cells. T cells were transfected as described in Material and Methods section, and studied for presence of p21 mRNA and protein expression. **A: p21 mRNA expression: **The expression of p21 mRNA is seen only in cells transfected with CD3Sp21 sense plasmid DNA (**lane 1**) compared to cells transfected with either CD3ASp21 plasmid DNA (**lane 2**) or the empty vector DNA (**lane 3**). An equal expression of β-actin, a house-keeping gene for these three representative cell lines is also shown. **B: p21 protein expression: **Comparisom of expression of p21 protein in the control DNA (**lane 1**), CD3Sp21 (**lane 2**) and CD3ASp21 (**lane 3**) transfected cells, is shown in Western blot analysis of p21 protein. **C: IL-2 mRNA expression: **The expression of Il-2 mRNA in cells transfected with CD3Sp21 DNA is almost inhibited (**lane 3**) compared to cells transfected with the empty vector DNA (**Lane 1**), where as IL-2 mRNA is increased in CD3Asp21 DNA transfected cells (**Lane 2**). An equal expression of β-actin, a house-keeping gene for these three representative cell lines is also shown for each transfection. CD3Sp21 = CD3sensep21 and CD3ASp21 = CD3Antisensep21 plasmid DNA

Transfection with CD3-p21 sense and antisense plasmid DNA in EL4 thyoma cell lines resulted in modulation of p21 gene expression. The results demonstrate that p21 mRNA expression was observed only in cells transfected with, CD3-p21 sense plasmid DNA but not in cells transfected with either empty vector (control) or CD3p21 antisense plasmid DNA (lane 3, Figure 3A). Similar pattern was observed with p21 protein expression (Figure 3B).

Efficacy of the transfection with CD3p21 sense and antisense plasmid DNA was assessed by mRNA expression of IL-2 in comparison with cells transfected with empty vector DNA. EL4-IL2, mouse T cells were transfected with DNA from either sense or antisense CD3p21 constructs using lipofectamine2000 (Gibco, Ling Island, USA). After 48 h, the cells were harvested, RNA was prepared and the expression of IL-2 was studied by RT-PCR and compared with untreated cells. The results in Figure 3C demonstrate that in comparison with empty vector DNA transfected cells (**Control**), the expression of IL-2 mRNA increased in cells transfected with anti-sense (**CD3ASp21**) and decreased in EL4-IL-2 cells treated with and sense plasmid DNA (**CD3Sp21**). The expresion of the house keeping gene β-actin in these cells was identical. Interestingly, the cells from CD3 Antisensep21 plasmid DNA transfected proliferated more and CD3Sensep21 plasmid DNA transfected proliferated less compared to untreated EL4-IL2 cells. These results demonstrate that it was feasible to modulate p21 gene expression in T cell line using CD3p21 chimeric constructs. As a control, human adenocarcinoma cells (A-549) and aortic smooth muscle cells were also transfected with CD3p21 sense plasmid DNA and did not result in increased p21 expression (data not shown).

### Effect of Targeted p21 modulation on mitogen induced T cell proliferation and T cell growth factor (IL-2) mRNA expression

**Figure 4:**
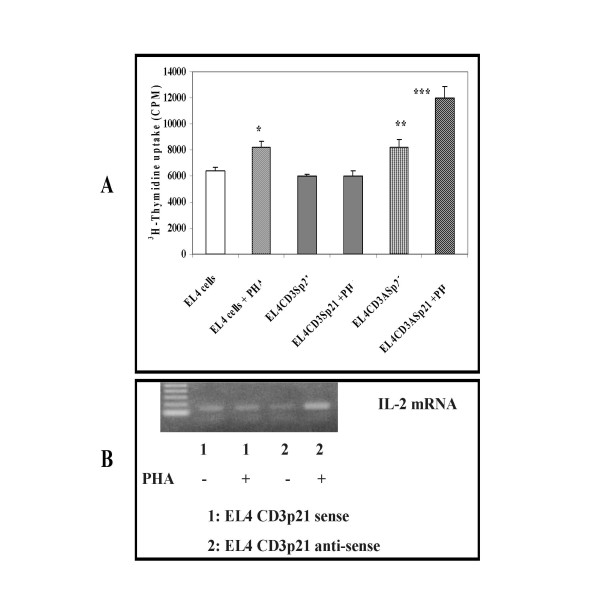
**Effect of direct p21 gene transfer in T cells on mitogen induced proliferation**. Three cell lines each from cells transfected with CD3p21 sense and CD3p21 antisense plasmid DNA were cultured with and without PHA for 4 h for IL-2 mRNA expression and 72 h (last 16 h with ^3^H-thymidine) for the proliferation assay. The results (A) demonstrate that p21 overexpressing EL4 T cells (CD3Sp21) did not respond to the mitogenic stimulus, whereas cells transfected with antisense plasmid DNA (CD3ASp21) proliferated significantly higher compared to untreated cells. *** = p < 0.02 (EL4 CD3ASp21 cells control vs PHA), ** = p < 0.03 EL4CD3Sp21 PHA vs CD3Asp21 PHA) and * = p < 0.05 control EL4 cells vs PHA. The mitogenic response was reflected in IL-2 mRNA expression by these cells. There was no difference in IL-2 mRNA expression between untreated and cells cultured with PHA for CD3Sensep21 plasmid DNA transfected cells (B, lanes 1 with a and without PHA). In sharp contrast, in cells transfected with CD3Antisensep21 plasmid DNA, PHA treatment resulted in a significant increase in IL-2 mRNA expression compared to the untreated cells.

To study the effect of p21 modulation on mitogen induced proliferation of T cells, EL4-IL-2 cells transfected with CD3p21 sense and antisense plasmid DNA were activated with mitogen phytohemaglutinin (PHA). The proliferation was quantified by ^3^H-thymidine uptake assay and IL-2 expression was studied by RT-PCR after treatment of these cells with PHA for 4 hours. The results shown in Figure 4A demonstrate the inability of EL4-IL2 cells transfected with CD3Sensep21 plasmid DNA to proliferate in response to mitogenic stimulus with PHA. Interestingly, EL4IL-2 cells transfected with CD3Antisensep21 plasmid DNA proliferated significantly (p < 0.02) more compared to the empty vector transfected cells. These results indicate the ability of CD3p21 chimeric constructs to alter proliferation of mouse T cells, by altering p21 expression.

EL4IL-2 cells transfected with either CD3Sensep21 or CD3Antisensep21 constructs were treated with PHA (1 μg/ml) for 4 hours, RNA was isolated and expression of IL-2 mRNA was studied by RT-PCR. The results shown in the Figure 4B demonstrate the increase in IL-2 expression only in EL4IL2 cells transfected with CD3Antisensep21 plasmid DNA. The increased IL-2 expression correlated with the proliferation of these cells in response to PHA.

### *In vivo *efficacy of CD3p21 sense and antisense plasmid DNA in mice

**Figure 5:**
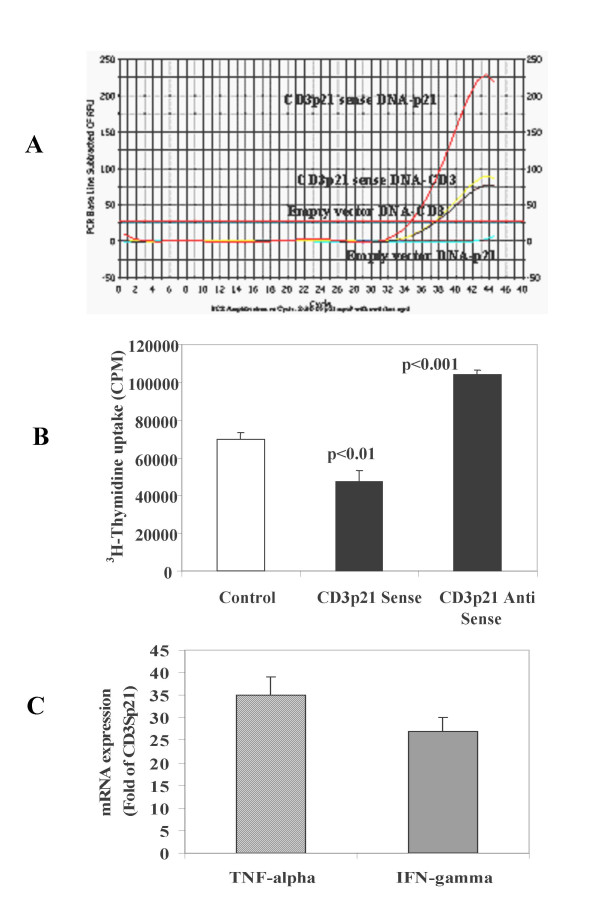
***In vivo *effects of direct transfer of p21 gene in mice**. **A: modulation of p21 gene expression: **Group of mice (n = 24) were injected intramuscularly with either empty vector, CD3p21 sense, CD3p21 Antisense plasmid DNA (n = 8 each). Modulation of p21 was monitored by p21 mRNA expression using Real Time PCR analysis of lymphocytes. Results from representative 2 animals from each group are shown. The mRNA expresion of both p21 and CD3 is seen only in mice injected with CD3p21 sense plasmid DNA, whereas as expected only CD3 mRNA expression is detectable in mice injected with empty vector plasmid DNA. These results demonstrate the ability of CD3p21 sense plasmid DNA to modulate p21 gene in T lymphocytes. **B: *In vivo *effect of p21 overexpression on lymphocyte proliferation**. Lymphocytes from mice injected with empty vector plasmid DNA (control mice n = 7), CD3p21 sense and antisense plasmid DNA (n = 7) were isolated and the proliferation in response to murine anti-CD3 monoclonal antibody was quantified using ^3^H-thymidine uptake assay. The lymphocytes from mice injected with CD3p21 sense plasmid DNA proliferated significantly (p < 0.01) less and with CD3p21 antisense plasmid DNA proliferated significantly (p < 0.001) more compared to the control mice. These results demonstrate that the direct gene modulation in T cells can influence their proliferation and inflammation. **C: *In vivo *effect of p21 modulation on TNF-α and IFN-γ mRNA expression: **RNA from lymphocytes obtained from mice injected with CD3Sp21 and CD3ASp21 plasmid DNA were isolated, reverse transcribed and analyzed for TNF-α and IFN-γ mRNA expression using Real Time PCR assay. Fold increase of mRNA expression in lymphocytes from CD3Antisensep21 injected mice was calculated with respect to the CD3Sensep21 DNA injected mice.

12 mice (C57/BL-6) were divided into three groups (n = 8 in each group). Mice were intramuscularly injected with either empty vector plasmid DNA, or CD3p21 sense or antisense plasmid DNA (100 μg). Mice were sacrificed after day 7 and splenocytes were prepared and activated with antiCD3 monoclonal antibody. First, we confirmed the presence of CD3 and p21 mRNA in lymphocytes from mice injected with CD3p21 sense or antisense plasmid DNA. The results (Figure 5A) demonstrate the presence of both CD3 and p21 mRNA in mice injected with CD3Sensep21 compared to only CD3 mRNA in mice injected with CD3Antisensep21 plasmid DNA. The results (Figure 5B) also indicate that splenocytes from mice transfected with CD3p21 mice proliferated significantly (p < 0.01) less than controls (Mean ± SEM 47264 ± 5805 vs 69729 ± 3333) and than mice transfected with CD3Asp21 plasmid DNA (104163 ± 2381, p < 0.001). The real time PCR analysis of these anti-CD3 activated lymphocytes demonstrated a TNF-α (35 fold) and IFN-γ (27 fold) increased mRNA expression in lymphocytes from CD3 antisensep21 plasmid DNA compared to CD3Sensep21 plasmid injected mice (Figure 5C).

## Discussion

The principal aim for this chimeric plasmid was to test the T cell targeted overexpression of p21 in T lymphocytes, since CD3 is surface marker for only T lymphocytes. We have earlier described the efficacy of p21 sense and antisense plasmid DNA to either overexpress or delete p21 gene in lymphoid and non-lymphoid cell lines [23]. Alloimmune activation remains a critical step in allograft rejection and survival. Therefore, a strategy, which could specifically target T lymphocytes, will provide an effective inhibition of alloimmune activation permitting prolonged graft survival. Our studies [5,23,24] have demonstrated that p21 is such an agent since it is a potent inhibitor of lymphocyte proliferation. Our studies have shown that its over expression not only renders lymphocytes less responsive to mitogenic and allogenic stimuli but also makes them more responsive to other anti-inflammatory agents such as CsA [23]. Our *in vitro *studies with another T cell line (Jurkat T cells) have also demonstrated that the overexpression of p21 resulted in their unresponsiveness to mitogenic stimuli [5,23]. These results demonstrate that p21 can be utilized as an effective agent to specifically target T lymphocytes to limit inflammation. Interestingly, mCD3delta promoter has been shown contributes largely to a T cell-specific expression pattern both in vitro and in transgenic mouse studies [1], supporting our studies with CD3promoter-p21 chimeric gene construct. Studies have also demonstrated that adenoviral vectors coupled to bispecific antibodies (bsAbs) with one of the antibodies to CD3 could target T cells that were normally resistant to adenoviral vectors. Similarly, surface-engineered lentiviral vectors significantly improved transduction of primary lymphocytes by activating the target cells [2]. But the results from these experiments explain the ability of a nonviral vector to directly transfer genes to T cell s with CD3 promoter gene construct.

A number of studies [1-4] have explored the efficacy of adenoviral or lentiviral vectors with different combinations of antibodies to MHC-1, T cell receptor (TCR)-CD3+ cytokine +chemokine to provide gene therapeutic approach for cancer treatment to bypass tumor immune mechanisms. However, in this study, the efficacy of a nonviral CD3p21 chimeric plasmid construct to effectively in vivo modulate p21 expression is provided. Previous studies have demonstrated that the overexpression of p21 induced by intramuscular injection of p21 sense plasmid DNA resulted in an increased graft survival in a rat heart transplant model [5]. The method of using plasmid DNA to obtain *in vitro *and *in vivo *transfection of p21 is based on the data supporting the efficacy of intra-muscular injection of plasmid DNA for a number of genes [25-27]. Intramuscular injection of naked plasmid DNA expression vectors encoding either TGF-beta1 (pVR-TGF-beta1) or an IL-4-IgG1 chimeric protein (pVR-IL-4-IgG1) resulted in production of TGF-beta1 or IL-4-IgG1, respectively, and protection from myelin basic protein (MBP)-induced experimental allergic encephalomyelitis (EAE) [28]. The *in vivo *specificity and efficiency of nonviral vectors has been demonstrated for IL-10 was found to be far more persistent than those achieved by viral vectors [29]. Similar results have been reported for a number of genes with a single intramuscular injection suggesting that the muscle tissue is accessible and expression is usually more persistent than elsewhere, vector administration is simple and the method is inexpensive. More significantly, plasmids do not induce neutralizing immunity, which permits repeated administration. One of the earlier criticisms of this methodology was that effect of gene modulation in immune cells might not be achieved but this and other studies [25-27] more significantly in human [30] demonstrates the feasibility of the gene transfer using plasmid DNA. Our method of *in vitro *and *in vivo *modulation of gene expression was very efficient.

Therefore, based on our published studies [5,20] and the results from this study, we demonstrate that the p21 over-expression obtained through direct gene transfer to T cells via intramuscular injections of CD3-p21 plasmid DNA results in the decreased responsiveness of T lymphocytes to mitogenic stimuli. Linearized DNA containing the CD3-p21 construct was used to overexpress p21 both *in vitro *(EL4-IL-2 cell line) and *in vivo *in mice (C57BL/6). These results uniquely describe the construction of a chimeric plasmid construct constituted of CD3 promoter and cyclin kinase inhibitor p21. The results demonstrate the feasibility of direct gene transfer to T cells with a CD3promoter-p21 chimeric construct.

## Conclusion

In summary, the results from this study uniquely describes the feasibilty of direct gene transfer into T cells with a chimeric CD3-p21 plasmid construct. The transfection of EL4 thymoma cells, which constitutively express IL-2 mRNA, using DNA with p21 in the sense and antisense direction resulted in modulation of T cell proliferation. The extent of cellular proloferation correlated with IL-2 mRNA expression. Most significantly, intramuscular injection of the CD3p21 sense and antisense plasmid DNA resulted in increased p21 expresion in T cells confirmed by the expression of CD3 and p21 mRNA expression. This overexpression resulted in unresponisveness of splenocytes from these mice to mitigen-induced proloferation. However, we did not explore the effect of p21 overexpression in subsets of T lymphocytes such as CD4 and CD8 cells, which may have different response. Furthermore, based on its effect on immunomodulation, it is likely that p21 plays an important role in genertaion and maintrenance of CD4+CD25+ T regulatoruy cells, which need to be explored. The ultimate goal of such a study is to utilize this methodology to target T cells to modulate gene expression in T cells responsible for site specific inflammation and alloimmune activation. The targeted overexpression of p21 in T lymphocytes to inhibit their proliferation and subsequently inflammation will provide the most suitable therapeutic strategy to inhibit alloimmune activstion in organ transplantation. This can only be accompalished using a gene therapeutic technique. Using various gene transfer technologies, the ability to transfer genes to the various cell types within whole animals has been describing allowing a wishful thinking to prevent and treat many diseases. It is therefore conceived that that the studies like this would pave the way to the development of such tools not just to treat disease but also to study the physiology of the healthy body. The ability to target tissue specific gene to alter the products (genes and proteins) responsible for a specific signaling or biosynthetic pathway, will be extremely beneficial. Thouogh these strategies seem possible however many hurdles and challenges that include efficient *in vitro *and *in vivo *transfection need to be overcome. But the studies like this if confirmed in a number of other models will provide an unique direction in the development of targeted gene therapeutic approaches to prevent not only only alloimmune activation in organ transplantation but also inflammtion in patients with cancer, arthritis, inflammatory bowel disease and others diseases associated with aberrant cellular proliferation like atherosclerosis.

## Methods

### Construction of CD3p21 chimeric constructs

Plasmid TGE-CD3 containing the murine T cell-specific delta enhancer and promoter was originally obtained from Dr Ronald Evans (Salk Institute, San Diego, CA). This CD3 promoter containing plasmid has successfully been shown to target genes specifically to T cells [31]. The enhancer region was isolated from the plasmid following restriction digestion with BamHI and cloned into the BamHI site of the pBluescript SK vector (Stratagene, La Jolla, CA). The murine CD3 promoter was cut with Pst I and blunted with T4 DNA polymerase. HindIII linkers were ligated to the fragment and, following HindIII digestion, cloned into the HindIII site downstream of the enhancer region. The p21 gene was excised from the zeo+-p21 plasmid construct [23] with restriction enzymes KpnI and XbaII, blunted with DNA polymerase I, large (Klenow) fragment, and cloned into the blunted Cla I site just downstream of the CD3 promoter in the enhancer/promoter SK construct. Proper orientation of all components was confirmed by sequence analysis. The transgene was then separated from plasmid sequences following restriction enzyme digestion, agarose gel electrophoresis, and electro elution. The presence of p21 gene and its orientation (sense and antisense) was confirmed by DNA sequencing. A number of clones expressing p21 gene in the sense or anti-sense orientation were obtained and termed as CD3 Antisensep21 and CD3Sensep21 constructs. Plasmid DNA from these clones was prepared and the presence of p21 gene was ascertained by separating restriction enzyme digested DNA on agarose gels.

### EL4-Thymoma cells Proliferation Assay

Cell proliferation was determined using ^3^H-thymidine incorporation assay as previously described [5]. All assays were performed in triplicate. A total of 3 individual experiments investigating the proliferation of unaltered and p21 EL4-Thyoma cells were performed in un-stimulated and activated with PHA. Briefly, 200,000 cells were added to each well of a round bottom 96-well plate. PHA (2 μg/ml) was added to the wells, controls were without PHA. The cells were cultured for 64 h at 37°C in 95% air and 5% CO_2 _enriched environment. The cultures were pulsed with ^3^H Thymidine (1 μCi/well) for the last 16 h of incubation, cells were harvested and radioactivity counted using a scintillation counter. ^3^H-Thymidine uptake was expressed as the mean counts per minute of triplicate samples. The magnitude of EL4-Thyoma T cells proliferation from unaltered and p21-augmented cells was investigated at rest and following mitogen stimulation.

### *In vitro *transfection of p21WAF1/CIP1

EL4 cells were transfected with CD3Sensep21, CD3Antisensep21 DNA as described earlier [23]. The increased or decreased level of p21 mRNA and protein expression was determined by RT-PCR and Western blot, respectively [20,21]. Cells transfected with empty vector plasmid DNA were used as controls.

### *In vivo *transfection of p21WAF1/CIP1

Mice (C57BL/6) were obtained from Jackson Labs. 8–12 week old mice of either sex, mice (n = 24) were non-injected (n = 8), injected with empty (n = 8), vector or injected with p21 100 μg of sense plasmid DNA (n = 8) and p21 100 μg of antisense plasmid DNA (n = 8) complexed with lipofectamine. Mice were sacrificed 7 days after the last DNA injection. Lymphocytes were isolated and the spontaneous and anti-CD3 induced *ex vivo *proliferation was studied. The efficacy of gene transfer was monitored by real time PCR analysis of p21 and CD3 mRNA in splenocytes.

### *In vitro *and *in vivo *efficiency of Transfection

*In vitro *and *in vivo *efficiency of our method of transfection was assessed by transfection of EL4-IL-2 thyoma cells and mice with p21-GFP construct, respectively. The authenticity of p21-GFP construct is shown in Figure 2A. For i*n vitro *assay, cells were transfected with p21-GFP construct and cells were washed and the percent of cells positive for green fluorescence were counted using Fluorescence Activated Cell Sorter. Untransfected and cells transfected with empty plasmid DNA were used as controls. For *in vivo *efficiency of our method, 5 mice were injected (intramuscular) with 100 μg of p21-GFP DNA or empty vector plasmid DNA. After 7 days, mice were sacrificed and RNA was isolated from spleens, reverse transcribed to cDNA and amplified for p21 and GFP mRNA.

### Detection of mRNA by reverse transcription and polymerase chain reaction (PCR) in lymphocytes

Total RNA was isolated from lymphocytes with Trizol (Invitrogen, Carlsbad, CA) and tissues with SV RNA isolation kit (Promega, Madison, WI). Purity of RNA was confirmed by a ratio of 260/280 nm. 1 μg of RNA was reverse transcribed into cDNA using a superscript reverse transcription kit from Invitrogen (Carlsbad CA). The amplification of specific mRNA expression was achieved by polymerase chain reaction (PCR) using specific primer sequences for p21, β-actin, IL-2 are described by us [5]. The PCR products were resolved in 1% agarose gel electrophoresis, ethidium bromide stained specific bands were visualized under UV light and photographed. A cycle analysis was performed for each primer pair to select a cycle number for amplification for each gene studied.

### Detection of mRNA by Real Time PCR

We performed real-time quantitative RT-PCR for CD3, p21, TNF-α and IFN-γ mRNA using a Bio-Rad iCycler system (Bio-Rad, Hercules, CA). RNAs were isolated from renal tissues using a kit from Promega (Madison, USA) and reverse-transcribed into cDNAs by using a cDNA synthesis kit from invitrogen (Carlsbad CA). The specificity of primers (**CD3 **Sense 5'-TGCTCTTGGTGTATATCTCATTGC-3', antisense 5'-CAGA GTCTGCTTGTCTGAAGCTC-3'; **p21 **Sense 5'-TCACTGTCTTGTACCCTT GTGC-3', antisense 5'-GGCGTTTGGAGTGGTAGAAA-3'; **IFN-γ **Sense 5'-TCTGGAGGA ACTGGCAAAAG-3', antisense 5'-TTCAAGACTTCAAAGAGTC TGAGG-3'; **TNF-α **Sense 5'-GACAAGCCTGTAGC CCATGT-3', antisense 5'-TCTCAGCTCC ACGCCATT-3' and **β-actin **sense: 5'-CCCAGCAC AAT GA AGATCAA-3' and antisense 5'-CGAT CCACACGG AGTACTTG-3) was tested by running a regular PCR for 40 cycles at 95°C for 20 s and 60°C for 1 minute, and followed separating in ethidium bromide containing agarose gels. The real-time PCR was performed using a SYBR supermix kit (Bio-RAD), and running for 40 cycles at 95°C for 20 s and 60°C for 1 minute. The PCR efficiency was also examined by serially diluting the template cDNA and the melting curve data was collected to check the PCR specificity and proper negative controls were included in each assay. The mRNA level for each gene for each sample was normalized to β-actin mRNA and was presented as 2 [(Ct/β-actin – Ct/gene of interest)] as described [32].

### Data analysis

Differences between groups were determined using two-tailed unpaired T test with significance considered present at a p value of less than 0.05. Statistical analysis was performed using a software program from GraphPad Software, Inc., San Diego, CA 92121 USA. The results are expressed as M ± SEM.

## Authors' contributions

AKK conceived the research design and plan, performed experiments, and wrote the manuscript. MRM contributed to the planning of the experiments and writing of the experiments and other valuable suggestions. Both authors read and approved the final manuscript.
